# Electrochemical C–N coupling with perovskite hybrids toward efficient urea synthesis†

**DOI:** 10.1039/d1sc01467f

**Published:** 2021-04-12

**Authors:** Menglei Yuan, Junwu Chen, Yiling Bai, Zhanjun Liu, Jingxian Zhang, Tongkun Zhao, Qiaona Shi, Shuwei Li, Xi Wang, Guangjin Zhang

**Affiliations:** CAS Key Laboratory of Green Process Engineering, Beijing Key Laboratory of Ionic Liquids Clean Process, State Key Laboratory of Multiphase Complex Systems, Institute of Process Engineering, Chinese Academy of Sciences Beijing 100190 China zhanggj@ipe.ac.cn; Center of Materials Science and Optoelectronics Engineering, University of Chinese Academy of Sciences Beijing 100049 China; State Key Laboratory of Coal Conversion, CAS Key Laboratory of Carbon Materials, Institute of Coal Chemistry, Chinese Academy of Sciences Taiyuan 030001 China; SynCat@Beijing, Synfuels China Technology Co. Ltd. Huairou District Beijing 101407 China; Sino Shaanxi Nuclear Molybdenum Industry Co. Ltd. Tongguan 714300 China; Key Laboratory of Luminescence of Optical Information, Ministry of Education, Beijing Jiaotong University Beijing 100044 China; Chemistry and Chemical Engineering Guangdong Laboratory Shantou 515031 China

## Abstract

Electrocatalytic C–N coupling reaction by co-activation of both N_2_ and CO_2_ molecules under ambient conditions to synthesize valuable urea opens a new avenue for sustainable development, while the actual catalytic activity is limited by poor adsorption and coupling capability of gas molecules on the catalyst surface. Herein, theoretical calculation predicts that the well-developed built-in electric field in perovskite hetero-structured BiFeO_3_/BiVO_4_ hybrids can accelerate the local charge redistribution and thus promote the targeted adsorption and activation of inert N_2_ and CO_2_ molecules on the generated local electrophilic and nucleophilic regions. Thus, a BiFeO_3_/BiVO_4_ heterojunction is designed and synthesized, which delivers a urea yield rate of 4.94 mmol h^−1^ g^−1^ with a faradaic efficiency of 17.18% at −0.4 V *vs.* RHE in 0.1 M KHCO_3_, outperforming the highest values reported as far. The comprehensive analysis further confirms that the local charge redistribution in the heterojunction effectively suppresses CO poisoning and the formation of the endothermic *NNH intermediate, which thus guarantees the exothermic coupling of *N=N* intermediates with the generated CO *via* C–N coupling reactions to form the urea precursor *NCON* intermediate. This work opens a new avenue for effective electrocatalytic C–N coupling under ambient conditions.

## Abstract 2

The local charge redistribution in BiFeO_3_/BiVO_4_ hybrids promotes the targeted adsorption and activation of inert gas molecules and guarantees the exothermic coupling of *N=N* with generated CO *via* C–N coupling reactions to form *NCON* precursor.

## Graphical abstract

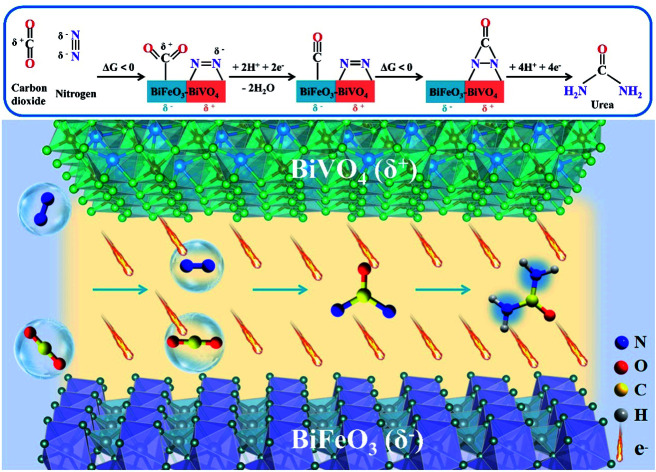

## 1 Introduction

Nitrogen (N_2_), accounting for 78% of the atmosphere, exists in the gaseous form that can't be directly utilized in biology and chemistry fields.^1–3^ On the other hand, carbon dioxide (CO_2_) generated from industries and transportation is the principal greenhouse gas causing serious environmental concerns.^4–8^ Thus, converting N_2_ and CO_2_ into value-added fuels and chemical products *via* the C–N coupling reaction is a promising approach for not only mitigating environmental issues and energy crisis but also high-value utilization of N_2_ and CO_2_.^9–12^ However, the highly stable double-bond (C=O, 806 kJ mol^−1^) in CO_2_ molecules and the triple-bond (N≡N, 940.95 kJ mol^−1^) in N_2_ molecules make the inert gas molecules difficult to activate mildly.^13–17^ Although conventional industrial processes such as the Haber–Bosch method and carbon capture and sequestration achieve the activation of inert gas molecules, their further utilization is impeded by the large energy consumption and complex synthetic processes.^18–22^ In comparison, energy-saving and environmentally benign electrocatalysis technologies such as the nitrogen reduction reaction (NRR) and the carbon dioxide reduction reaction (CO_2_RR) are drawing growing attention.^23–30^ Besides, the emerged electrochemical C–N coupling reaction may provide a new possibility of enhancing the spectrum of products of CO_2_ by using CO_2_ and amine derivatives/nitrogen sources (nitrate, nitrite, NO, and even N_2_) as feedstock.^9,31,32^

The desired product urea [CO(NH_2_)_2_] will be produced when the electrochemical C–N bond formation occurs by employing both N_2_ and CO_2_ as the feeding gas.^9^ Urea is commonly utilized as the general feedstock in industry and the primary fertilizer for agriculture.^33,34^ The industrial urea synthesis proceeds by two consecutive processes, including N_2_ + H_2_ → NH_3_ followed by NH_3_ + CO_2_ → CO(NH_2_)_2_, which operate under harsh reaction conditions (350–550 °C, 150–350 bar and 150–200 °C, 150–250 bar, respectively).^35^ Compared with the complex industrial synthetic process, simultaneous electrocatalytic fixation of N_2_ and CO_2_ driven by a renewable energy source under ambient conditions provides a clean route for urea production.^9^ The main issues in electrochemical synthesis of urea lie in three aspects: (1) extraordinarily weak chemical adsorption of inert CO_2_/N_2_ on the catalysts surface;^29,30,36,37^ (2) the dissociation of the highly stable C=O bond and N≡N bond requires high overpotential;^13,15^ (3) the parallel reaction of CO_2_/N_2_ reduction suppresses the efficiency of C–N coupling and strongly competes with the desired urea formation reaction, further resulting in a large distribution of complex products.^9^

Although a few noble metal Pd-based catalysts can achieve urea electrosynthesis, the related catalytic activity showed a maximum urea yield rate of 3.36 mmol h^−1^ g^−1^ with a FE of 8.92%.^9^ Besides, the high price and scarcity of the used noble metals impede their real application on the large scale. Thus, further improvement of the catalytic activity with earth-abundant materials remains a challenge. Inspired by the different electronic structures of N_2_ and CO_2_ molecules, tuning the electronic state of the electrocatalyst's surface can be a feasible strategy to optimize the adsorption of reactant gas molecules and suppress the competing electro-reduction reactions to promote urea generation. In this regard, fabricating a built-in electronic field to accelerate the local charge redistribution in perovskite heterostructures displays fascinating potential to deliver impressive urea electroproduction activity. On the one hand, perovskite structured transition-metal oxide (ABO_3_) semiconductors possess a distinctive electronic structure, which can lead to the alteration of electron density when combining with other domains.^38,39^ On the other hand, the work function difference in perovskite heterostructures drives the local charge redistribution by the band bending at the heterointerface, which is preferred for targeted adsorption and activation of inert small molecules such as N_2_, CO_2_, H_2_O, and so on.^40^ Despite the encouraging merits, inducing the local charge redistribution in perovskite hybrids toward the specific urea electrosynthesis hasn't been explored.

In this work, with the aid of theoretical simulation, we exploited perovskite structured p-type BiFeO_3_ and an n-type semiconductor BiVO_4_ to fabricate innovative p–n heterojunction electrocatalysts, aiming at enabling spontaneous electron transfer at the heterointerfaces by the desirable built-in electric field. The obtained perovskite structured BiFeO_3_/BiVO_4_ showed high electrocatalytic activity toward the C–N coupling reaction to synthesize urea, and exhibits a FE of 17.18% in 0.1 M KHCO_3_ at −0.4 V *vs.* RHE. The urea yield rate can reach 4.94 mmol h^−1^ g^−1^ which is much higher than the recently reported best values with Pd-based electrocatalysts. The activity can further be improved by adding an ionic liquid to the electrolyte. The generated local electrophilic and nucleophilic regions enhanced the targeted adsorption and activation of inert N_2_ and CO_2_ molecules and balanced the competing electro-reduction reactions to further promote the formation of the *NCON* intermediate *via* the C–N bond coupling reaction. Thus, engineering a built-in electric field to facilitate local charge redistribution has been proposed as an appealing strategy to enhance electrocatalytic C–N bond coupling and further urea synthesis.

## 2 Results and discussion

**Fig. 1:**
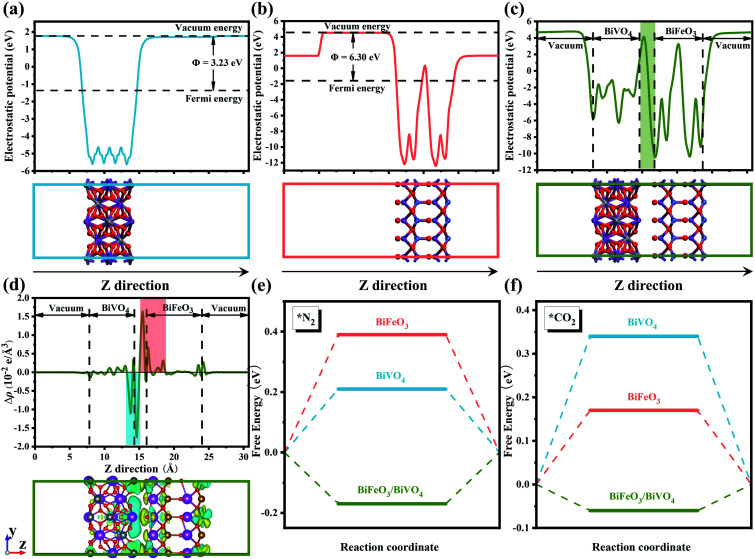
The calculated electrostatic potentials and work functions for (a) BiVO_4_, (b) BiFeO_3_ and (c) BiFeO_3_/BiVO_4_ heterojunction surfaces; (d) planar average charge density difference along the *z*-direction for the BiFeO_3_/BiVO_4_ heterojunction, the bottom image shows the charge density difference of the BiFeO_3_/BiVO_4_ heterojunction, the yellow and cyan color indicate electron accumulation and depletion, respectively, with an isosurface value of 0.013 e Å^−3^; free energy diagrams for (e) N_2_ and (f) CO_2_ adsorption on BiFeO_3_, BiVO_4_ and the BiFeO_3_/BiVO_4_ heterojunction.

Conspicuously, the charged surface can give rise to a significant effect on the targeted adsorption of reactant molecules.^30^ In this regard, constructing built-in fields at the heterointerface to promote a spontaneous electron transfer process will supply more possibilities for generating desirable two opposite charged regions.^41^ This design concept has been confirmed by the investigation of various perovskite-based semiconductor catalysts, which can constitute distinct p–n junctions and thus accelerate local charge redistribution at the heterointerface.^40^ The band theory of solids has confirmed that the behavior of electron transfer is strongly correlated to the work function of semiconductors.^42^ As presented in Fig. 1a and b, the theoretical simulation results showed that the work function values of BiVO_4_ and BiFeO_3_ were 3.23 eV and 6.30 eV, respectively, which allow self-driven electron transfer from BiVO_4_ to BiFeO_3_. The profile of the planar averaged electrostatic potential along the *z*-direction for BiFeO_3_/BiVO_4_ hybrids is depicted in Fig. 1c. Compared with pristine BiVO_4_ and BiFeO_3_ that possess periodic lattice potential, BiFeO_3_/BiVO_4_ hybrids showed a big build-in potential at the interfaces. Additionally, electron density difference calculation was further performed to reveal the charge distribution at the interface of BiFeO_3_/BiVO_4_ hybrids. As described in Fig. 1d, the charge depletion and accumulation are represented by the cyan region and yellow region, respectively. An increase of the charge density was observed at the BiFeO_3_ surface and a decrease at the BiVO_4_ surface, which confirmed that the spontaneous electron transfer induced the local charge redistribution around the interface and endowed the surfaces of BiFeO_3_ and BiVO_4_ with local nucleophilic and electrophilic regions. Bader analysis results further demonstrate that BiVO_4_ can transfer 2.33 electrons to BiFeO_3_. Previous research has proven that the chemisorption of inert CO_2_ and N_2_ is the initial step for electrocatalytic urea production. As shown in Fig. S1,† the N atom in the N_2_ molecule and the C atom in the CO_2_ molecule are electron-rich and electron-deficient, respectively. Therefore, it can be deduced that the rationally designed BiFeO_3_/BiVO_4_ hybrids with local nucleophilic and electrophilic regions would adsorb targeted reactant molecules by electrostatic interaction. To confirm the above hypothesis, density functional theory (DFT) calculations were performed to reveal the gas adsorption behavior on the surface of the electrocatalyst. As expected, the calculated adsorption energies of BiFeO_3_/BiVO_4_ hybrids for N_2_ and CO_2_ are −0.17 eV and −0.06 eV, much lower than those of individual architectures of BiFeO_3_ and BiVO_4_ (Fig. 1e and f), proving that N_2_ and CO_2_ show a stronger tendency to adsorb on the surface of BiFeO_3_/BiVO_4_ hybrids than on pristine BiFeO_3_ and BiVO_4_ due to the interfacial interaction triggered by the well-defined space-charge region, which is favorable for much readily chemisorbing inert gas molecules and thus facilitating the electrocatalytic urea production process.

**Fig. 2:**
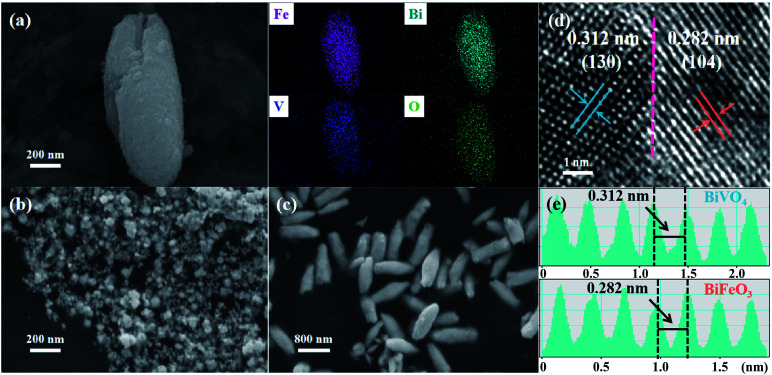
(a) SEM image and the corresponding elemental mapping of BiFeO_3_/BiVO_4_ hybrids; SEM images of (b) BiFeO_3_ and (c) BiVO_4_; (d) high-resolution TEM image of BiFeO_3_/BiVO_4_ hybrids and the dotted line represents the heterointerfaces; (e) the well-resolved lattice fringe of BiFeO_3_/BiVO_4_ hybrids in Fig. 2d.

As a proof-of-concept experiment, perovskite structured BiFeO_3_/BiVO_4_ hybrids were designed and synthesized by the facile ultrasonic bath method. The field-emission scanning electron microscopy (FE-SEM) image in Fig. 2a reveals that BiFeO_3_/BiVO_4_ heterostructures possess a rice-like morphology with an average length of 1 μm and a diameter of approximately 400 nm, respectively. The relevant elemental mapping demonstrates the evenly distributed Bi, Fe, V, and O elements. In comparison, pristine BiVO_4_ still maintains the same morphology as heterostructured hybrids (Fig. 2c), whereas the BiFeO_3_ displays an irregular nanoparticle structure (Fig. 2b). As surveyed by high-resolution transmission electron microscopy (HR-TEM), the well-resolved lattice fringes of 0.282 nm and 0.312 nm corresponded to the (104) plane and (130) plane of BiFeO_3_ and BiVO_4_ crystals (Fig. 2e). Meanwhile, the distinct interface generated by the intimate contact of BiFeO_3_ and BiVO_4_ confirms the representative establishment of nanoscale heterostructures (Fig. 2d).

**Fig. 3:**
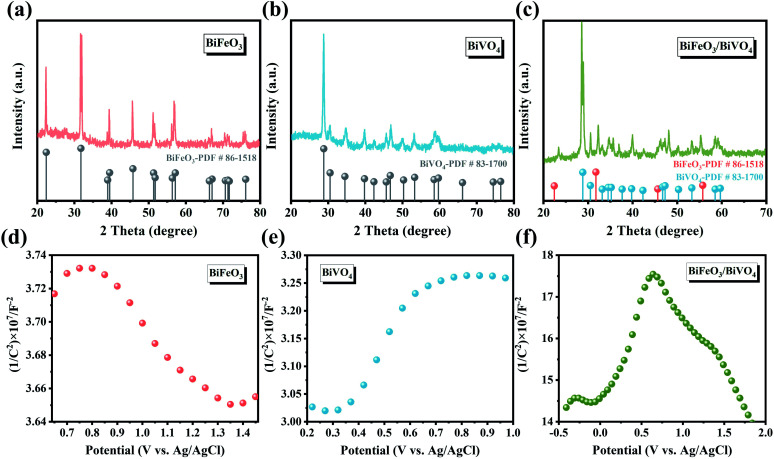
XRD patterns of (a) BiFeO_3_, (b) BiVO_4_ and (c) BiFeO_3_/BiVO_4_; Mott–Schottky plots of (d) BiFeO_3_, (e) BiVO_4_ and (f) BiFeO_3_/BiVO_4_.

**Fig. 4:**
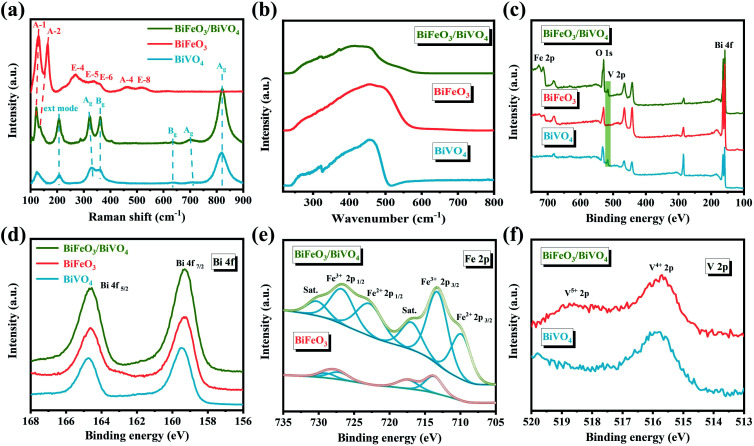
(a) Raman spectra, (b) UV-Vis spectra, (c) XPS survey spectrum and (d) high-resolution Bi 4f spectrum of BiFeO_3_, BiVO_4_ and BiFeO_3_/BiVO_4_; (e) high-resolution Fe 2p spectrum of BiFeO_3_ and BiFeO_3_/BiVO_4_; (f) high-resolution V 2p spectrum of BiVO_4_ and BiFeO_3_/BiVO_4_.

In Fig. 3a and b, the XRD spectra show that the hexagonal BiFeO_3_ structure (JCPDS: 86-1518) and monoclinic BiVO_4_ phase (JCPDS: 83-1700) can be obtained in pristine BiFeO_3_ and BiVO_4_ samples. Concerning BiFeO_3_/BiVO_4_ hybrids, both sets of diffraction peaks are consistent with those of pristine samples, except for the slight shift of the characteristic peaks of BiFeO_3_ and partial peaks of BiVO_4_ to higher diffraction angles (Fig. 3c), which indicates the possible interaction between the two distinct domains.^43,44^ As further revealed by Mott–Schottky (M–S) curves, BiFeO_3_ with a negative slope (Fig. 3d) and BiVO_4_ with a positive slope (Fig. 3e) matched well with the apparent characteristics of a p-type and an n-type semiconductor, respectively. In comparison, the as-prepared BiFeO_3_/BiVO_4_ hybrids exhibited evident p–n heterojunction features (Fig. 3f), which furnish the rational architecture to achieve the desired local charge redistribution by the above theoretical prediction. In Raman spectra (Fig. 4a), the typical vibrational bands at 137 and 171 cm^−1^ represent the Fe–O–Fe bonds of the BiFeO_3_ sample,^45,46^ whereas they exhibit a slightly negative shift accompanied by an intensity decrease when coupled with BiVO_4_ domains. Likewise, the detected V–O stretching modes (637, 702, and 826 cm^−1^) and VO_4_^3−^ tetrahedron bending modes (327 and 367 cm^−1^) also negatively shifted after the establishment of the heterostructure with the space-charge region.^47–49^ This broadening of vibrational modes and position shifting in BiFeO_3_/BiVO_4_ hybrids manifest the intense coupling interaction between BiFeO_3_ and BiVO_4_.^50,51^ Besides, compared to the UV-Vis spectrum of pristine samples, the dominant peak in BiFeO_3_/BiVO_4_ hybrids exhibited an obvious blue shift, evidencing the existence of the charge transfer effect (Fig. 4b).^52^ To further reveal the electronic effects between BiFeO_3_ and BiVO_4_, the chemical components and the alteration of valence states in the formed catalysts were further examined by X-ray photoelectron spectroscopy (XPS). The survey spectra, displayed in Fig. 4c, illustrate the presence of Bi, Fe, V, and O elements in the obtained electrocatalysts, which is consistent with the above SEM characterization. The high-resolution Bi 4f spectrum displayed two predominant peaks of Bi^3+^ 4f_7/2_ and Bi^3+^ 4f_5/2_ at the binding energies of 159.3 eV and 164.7 eV (Fig. 4d).^53,54^

Besides, two distinct peaks centered at 713.4 eV and 726.9 eV in the Fe 2p region can be attributed to the binding energies of Fe^3+^ 2p_3/2_ and Fe^3+^ 2p_1/2_, respectively (Fig. 4e).^55,56^ And the XPS peak positioned at 515.8 eV in the V 2p spectrum illustrates the presence of V^4+^ (Fig. 4f). It is noteworthy that when BiFeO_3_ was coupled with BiVO_4_, the emerged new peaks in BiFeO_3_/BiVO_4_ hybrids are assigned to Fe^2+^ 2p and V^5+^ 2p peaks in contrast to pristine samples, elucidating that the apparent electronic interactions between BiFeO_3_ and BiVO_4_ domains are due to the formation of p–n heterojunctions.^57,58^ More importantly, the related changes in the valence state further demonstrate that the charge transferred from BiVO_4_ to BiFeO_3_. All these results convincingly suggest the successful establishment of unique p–n heterojunctions and the transfer of electrons between BiVO_4_ and BiFeO_3_. The induced local charge redistribution contributes to the targeted adsorption of reactant molecules and thus enhances the electrocatalytic urea production ability.

**Fig. 5:**
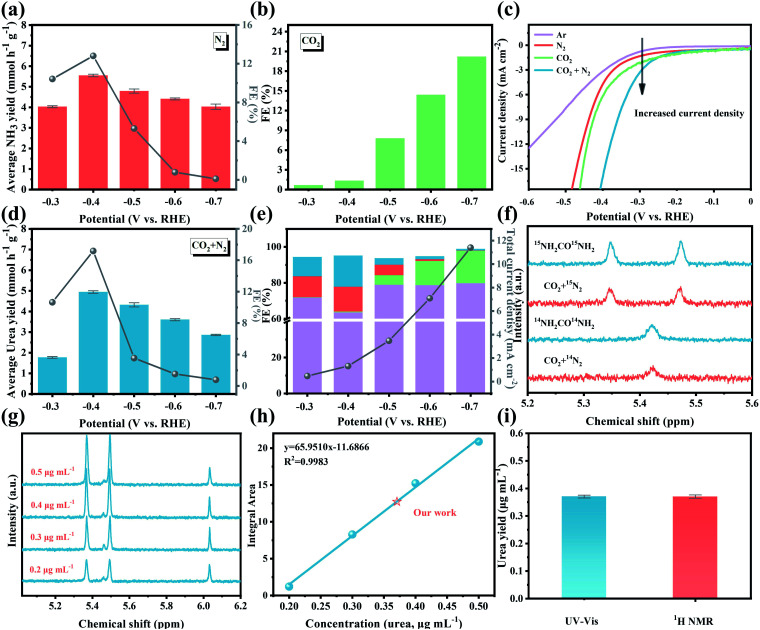
(a) NH_3_ synthesis with N_2_ as the feed gas and (b) CO generation with CO_2_ as the feed gas at various potentials for BiFeO_3_/BiVO_4_ hybrids; (c) LSV curves of BiFeO_3_/BiVO_4_ hybrids in Ar, N_2_, CO_2_ and N_2_ + CO_2_ saturated electrolyte; (d) the urea yield rate and faradaic efficiencies and (e) the corresponding product distribution of H_2_ (purple color), CO (cyan-blue color), NH_3_ (red color) and urea (blue color) for urea production with N_2_ and CO_2_ as the feed gas at various potentials for BiFeO_3_/BiVO_4_ hybrids; (f) ^1^H NMR spectra of electrolyte saturated with ^15^N_2_ + CO_2_/^14^N_2_ + CO_2_ after 2 h of electrolysis and standard ^15^NH_2_CO^15^NH_2_/^14^NH_2_CO^14^NH_2_ solution; (g) ^1^H NMR spectra of standard ^15^NH_2_CO^15^NH_2_ solution at concentrations of 0.2–0.5 μg mL^−1^; (h) integral area (^15^NH_2_CO^15^NH_2_/C_4_H_4_O_4_)–concentration linear relation calibrated using standard ^15^NH_2_CO^15^NH_2_ solution; (i) the urea yield of BiFeO_3_/BiVO_4_ hybrids after 2 h of electrolysis determined using the UV-Vis spectrum and ^1^H NMR spectra.

The electrocatalytic activity of BiFeO_3_/BiVO_4_ hybrids for the C–N coupling reaction toward urea production was tested in a 0.1 M KHCO_3_ solution utilizing an H-type two-compartment cell separated by a Nafion 211 membrane, which is equipped with a three-electrode configuration (Fig. S2†). Ultrahigh purity CO_2_ gas (99.999%) and N_2_ gas (99.999%) were continuously purged into the cathodic chamber with the same flow rate of 5 mL min^−1^ during the electrolysis process. The possibly generated liquid products (urea and NH_3_) in the electrolytes were spectrophotometrically measured by the diacetyl monoxime method and indophenol blue method.^22,59^ Meanwhile, the related calibration curves are displayed in Fig. S3.† Besides, online gas chromatography monitored the possible gas products such as CO and H_2_. It has been reported that the electrochemical catalytic activity of the C–N coupling reaction for urea production was dominant as a result of effectively coupling the carbon dioxide reduction reaction (CO_2_RR) with the nitrogen reduction reaction (NRR). As displayed in Fig. 5a and b, the BiFeO_3_/BiVO_4_ hybrids possess a high NH_3_ faradaic efficiency (FE) (12.81%) for the NRR and CO FE (20.21%) for the CO_2_RR, which establish the baseline of N_2_ and CO_2_ reduction activity. The linear sweep voltammetry curves (LSV) were initially examined in 0.1 M KHCO_3_ saturated with different gas feeds (Ar, CO_2_, N_2_, or CO_2_ + N_2_). As depicted in Fig. 5c, the distinctly enhanced current density in CO_2_ + N_2_ saturated electrolyte relative to that in CO_2_ and N_2_, respectively, indicates the occurrence of the electrocatalytic C–N coupling reaction for urea production. A potentiostatic experiment was further performed to quantitatively assess the performance of electrocatalytic urea production of BiFeO_3_/BiVO_4_ hybrids at different working potentials. As shown in Fig. S4,† the corresponding chronoamperometry curves within the potential range of −0.3 V to −0.7 V exhibit stable current density after electrolysis for 2 h. As demonstrated in Fig. 5d, the urea yield rate and the corresponding FE increase with the increase of applied potential. The highest urea yield rate is 4.94 mmol h^−1^ g^−1^ with a FE of 17.18% at −0.4 *vs.* the reversible hydrogen electrode (RHE), outperforming all the reported values for Pd based catalysts (Table S1†). However, at more negative potentials, the electrocatalytic urea production performance decreases, which may result from the occupation of the active sites for N_2_ and CO_2_ reduction by the excessively released CO (Fig. 5e). Additionally, the as-obtained BiFeO_3_/BiVO_4_ hybrids retain 96% of the initial current density after a 10 h long-term chronopotentiometry test (Fig. S5†). Likewise, when conducting five cycling tests, negligible change is observed in the electrocatalytic urea production performance (Fig. S6†), attesting to the superior electrocatalytic stability of BiFeO_3_/BiVO_4_ hybrids. The corresponding characterization studies further reveal that the BiFeO_3_/BiVO_4_ hybrids were well-matched with their original morphology, crystal phase, and chemical states after 10 h of continuous electrolysis (Fig. S7†), corroborating their robust structural stability. Impressively, with the aid of enhanced CO_2_ adsorption capacity of an ionic liquid,^60^ when the 1-butyl-3-methylimidazolium tetrafluoroborate–KHCO_3_ ([Bmim]BF_4_–KHCO_3_) electrolyte was used in the system, a higher electrocatalytic activity for urea production is achieved (FE: 20.75%, urea yield rate: 5.42 mmol h^−1^ g^−1^) than that obtained in pristine KHCO_3_ solution at the same potential (Fig. S8†).

To gain solid proof that the produced urea originated from the C–N coupling reaction with CO_2_ and N_2_, a rigorous protocol was employed to avoid false-positive results caused by the contamination of environmental NO_*X*_ (Fig. S9†). Under all the control experiment conditions, negligible urea was detected, which excludes the possible effect of environmental NO_*X*_ on the urea electrosynthesis (Fig. S10†). ^15^N_2_ isotopic labeling experiment was further utilized to corroborate the N source of the generated urea [CO(NH_2_)_2_]. The standard CO(^15^NH_2_)_2_ sample displays two dominant peaks at approximately 5.35 ppm and 5.47 ppm in ^1^H Nuclear Magnetic Resonance (NMR) (Fig. 5f), while the standard CO(^14^NH_2_)_2_ sample possesses one distinct peak at about 5.42 ppm. When utilizing ^15^N_2_ and CO_2_ as the feed gas, the detected ^1^H NMR signals of the produced urea matched well with the standard CO(^15^NH_2_)_2_ signals. Additionally, the concentration of CO(^15^NH_2_)_2_ was also quantitatively detected by ^1^H NMR, and the related signal integration–concentration linear relation is exhibited in Fig. 5g and h. As expected, the calculated urea concentration is consistent with the quantitative results of the diacetyl monoxime method (Fig. 5i). All these results are convincingly indicative of urea originating from the C–N coupling reaction from N_2_ and CO_2_ catalyzed by BiFeO_3_/BiVO_4_ hybrids.

**Fig. 6:**
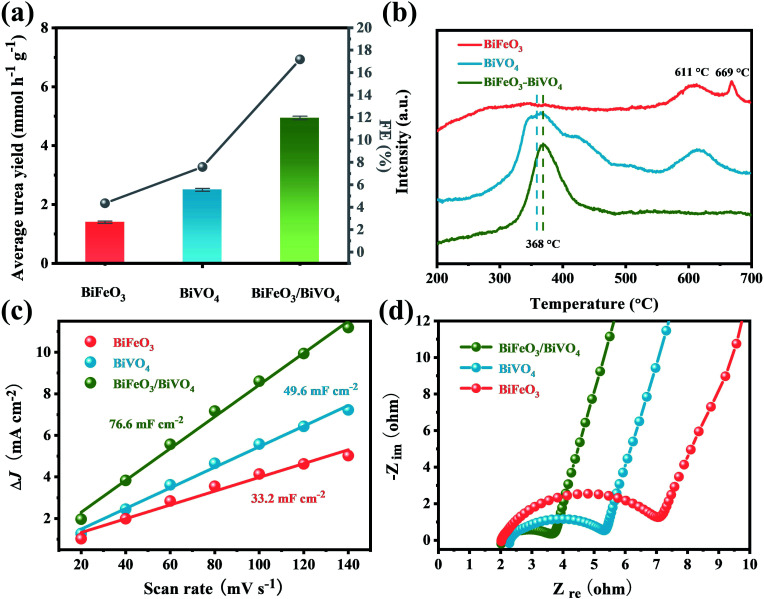
(a) Average urea yield; (b) carbon dioxide temperature-programmed desorption (CO_2_-TPD) spectra; (c) Δ*J* of electrocatalysts plotted against scan rate at −0.05 V *vs.* RHE; (d) Nyquist plots of electrochemical impedance spectra (EIS) of BiFeO_3_, BiVO_4_ and BiFeO_3_/BiVO_4_.

For comparison, the pristine BiFeO_3_ and BiVO_4_ were also evaluated under identical conditions. At the optimal potential of −0.4 V, the electrocatalytic activities of BiFeO_3_ (urea yield rate: 1.41 mmol h^−1^ g^−1^, FE: 4.35%) and BiVO_4_ (urea yield rate: 2.50 mmol h^−1^ g^−1^, FE: 7.59%) were much inferior to those of BiFeO_3_/BiVO_4_ hybrids for urea production (Fig. 6a). This indicates that the local charge redistribution in BiFeO_3_/BiVO_4_ hybrids plays a critical role in enhancing electrocatalytic urea production. As revealed by temperature-programmed desorption (TPD), compared with pristine BiFeO_3_ and BiVO_4_, BiFeO_3_/BiVO_4_ hybrids exhibited stronger binding strength and larger desorption peak in the CO_2_- and N_2_-TPD spectra (Fig. 6b and S11†), elucidating that the local charge redistribution in BiFeO_3_/BiVO_4_ hybrids endows the surfaces of BiFeO_3_ and BiVO_4_ with local nucleophilic and electrophilic regions and thus promotes the targeted adsorption of CO_2_ and N_2_ molecules, which is in good agreement with the aforementioned theoretical prediction. The electrochemically active surface area (ECSA) results suggest that the BiFeO_3_/BiVO_4_ hybrids (76.6 mF cm^−2^) display a higher electrochemical double-layer capacitance (*C*_dl_) than pristine BiFeO_3_ (33.2 mF cm^−2^) and BiVO_4_ (49.6 mF cm^−2^), which signifies that local charge redistribution promotes the exposure of more active sites in BiFeO_3_/BiVO_4_ hybrids for gas molecule adsorption and activation (Fig. 6c and S12†). Electrochemical impedance spectroscopy (EIS) analysis further reveals that the BiFeO_3_/BiVO_4_ hybrids possess a smaller semicircle and higher slope than pristine samples, evincing that the presence of local charge redistribution in BiFeO_3_/BiVO_4_ hybrids significantly promotes electron/ion transfer kinetics during the electrocatalytic process (Fig. 6d).^29,43^

**Fig. 7:**
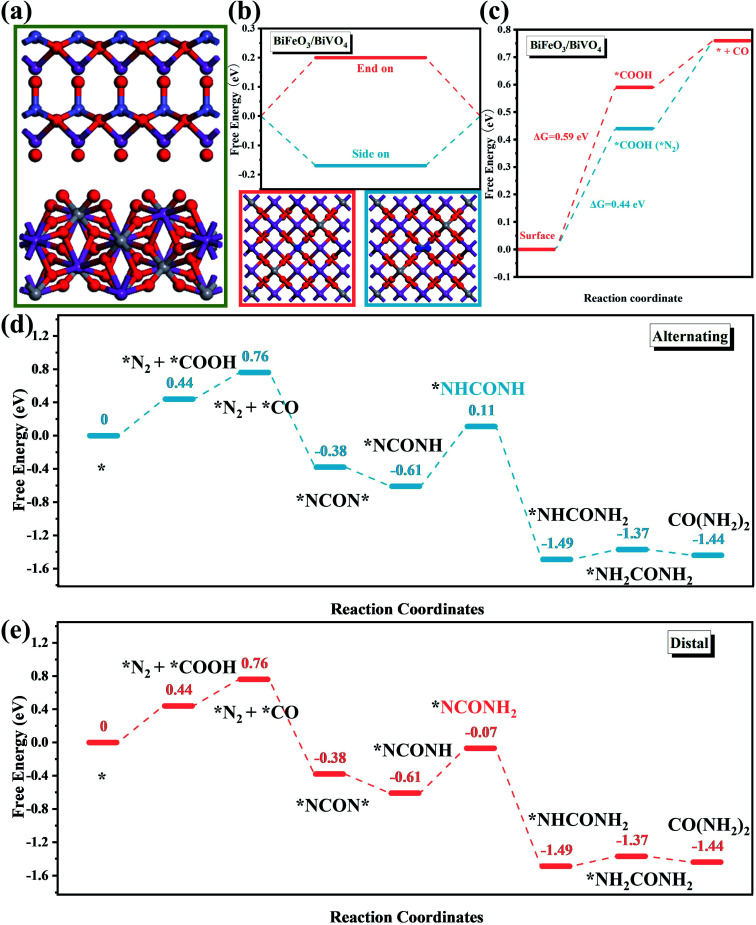
(a) The calculation model of the BiFeO_3_/BiVO_4_ heterostructure; free energy diagrams for (b) N_2_ adsorption and (c) CO_2_ reduction with and without N_2_ adsorption on the BiFeO_3_/BiVO_4_ heterostructure; the electrolytic urea production *via* (d) alternating and (e) distal mechanisms.

To obtain deeper insight into the C–N coupling reaction mechanism toward the electrocatalytic urea production, DFT calculation was further employed to assess the intermediate variation and energy barrier during the reaction process. The corresponding BiFeO_3_/BiVO_4_ heterostructured architecture is displayed in Fig. 7a. Since the chemisorption of inert CO_2_ and N_2_ molecules is the initial step for electrocatalytic urea production, it is critical to reveal the competitive adsorption between these gas molecules. In comparison with CO_2_-TPD, the peaks in the N_2_-TPD spectrum of the heterostructured hybrids appeared at a higher temperature and exhibited enhanced intensity, demonstrating that the BiFeO_3_/BiVO_4_ hybrids possessed stronger N_2_ chemisorption ability than that for CO_2_ molecules (Fig. 6c and S11†).^9^ In other words, the C–N coupling reaction was initiated with chemisorption of N_2_ molecules on the BiFeO_3_/BiVO_4_ hybrids. Besides, the free energy of the N_2_ adsorption with a side-on configuration was more negative than that of an end-on configuration, suggesting that N_2_ preferably adsorbed on the local electrophilic BiVO_4_ regions *via* the more stable side-on configuration (Fig. 7b). Impressively, as identified in Fig. 7c, the BiFeO_3_/BiVO_4_ hybrids required the highest Gibbs free energy (Δ*G*) of 0.59 eV to reduce CO_2_, while the Δ*G* decreased to 0.44 eV when the N_2_ molecules emerged in neighboring BiVO_4_ regions. Therefore, it can be deduced that the adsorbed/activated N_2_ molecules would facilitate the reduction of adsorbed CO_2_ to CO on local nucleophilic BiFeO_3_ regions. Then the C–N coupling reaction spontaneously occurred between the activated N_2_ molecules (*N=N*) and *in situ* generated CO to form the *NCON* intermediate, due to the matching molecular orbitals. The corresponding Δ*G* further corroborates that the formation of the *NCON* intermediate is an exothermic process. Once the *NCON* intermediate is generated, the subsequent hydrogenation of *NCON* would produce two possible reaction pathways involving distal and alternating mechanisms. Concerning the alternating pathway, the (H^+^ + e^−^) alternately reacted with the two N atoms, while the protonation continuously attacked the distal N atoms for the distal mechanism. As displayed in Fig. 7d and e, when the reduction of *NCON* follows the distal pathway, the required Δ*G* decreased to 0.54 eV compared to that of the alternating pathway (0.72 eV), suggesting that the BiFeO_3_/BiVO_4_ hybrids were prone to pursue the distal mechanism until the release of urea molecules rather than the alternating pathway from the thermodynamic perspective.^31^

The selectivity of the electrocatalytic urea production is closely associated with the formation of *NCON* intermediates. The possible N_2_ reduction or the excessive release of CO would result in a decrease in the efficiency of the electrocatalytic C–N coupling reaction and further reduce the selectivity. On the one hand, the Δ*G* for the reductive protonation of *N_2_ into *N_2_H (N_2_ + H^+^ + e^−^ + * → *NNH), which is regarded as the potential-determining step (PDS) toward the NRR, was calculated to be 0.14 eV (Fig. S13†),^61^ which was much higher than that for the C–N coupling reaction to form *NCON*. Such a significantly larger energy barrier makes BiFeO_3_/BiVO_4_ hybrids inactive for the electrocatalytic NRR, let alone generating side product NH_3_. On the other hand, the release of the generated *CO intermediate was strongly associated with the selectivity of the electrocatalytic urea production. Thus, the stability of the as-prepared electrocatalysts against CO poisoning was evaluated through an electrochemical CO-stripping experiment. As shown in Fig. S14,† the CO-stripping peak of BiFeO_3_/BiVO_4_ hybrids (0.251 V *vs.* RHE) exhibits a more negative shift than that of pristine BiFeO_3_ (0.261 V *vs.* RHE) and BiVO_4_ counterparts (0.265 V *vs.* RHE), indicating that the BiFeO_3_/BiVO_4_ hybrids possessed higher stability against CO poisoning, which is due to the elimination of some of the strong adsorption sites by the local charge redistribution.^62^ Notably, the amount of CO should be well controlled. As confirmed by Fig. 5e, when the applied potential exceeded −0.4 V, the excessively released CO occupied the adsorption sites for N_2_ and CO_2_ and resulted in a remarkable decrease of the FE for urea production.

**Fig. 8:**
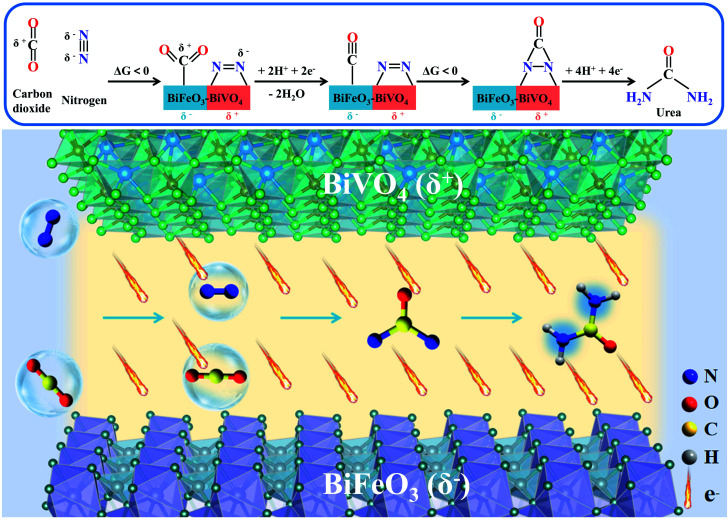
The schematic electrocatalytic urea production mechanism based on BiFeO_3_/BiVO_4_ p–n heterostructure synergistic effects.

By combining the aforementioned experimental results and computational simulations, the overall urea electrosynthesis process can be summarized in the following steps: (i) the built-in electric field in BiFeO_3_/BiVO_4_ hybrids accelerates the local charge redistribution, (ii) N_2_/CO_2_ molecules first adsorbed on the generated electrophilic/nucleophilic regions by electrostatic interaction, (iii) the produced *N_2_ promotes CO_2_ reduction under the electric field, and then the generated CO will further react with *N_2_ to produce the desirable *NCON* intermediate through exothermic electrocatalytic C–N coupling reaction, and (iv) the subsequent protonation process preferentially undergoes the distal mechanism until the formation of urea (Fig. 8).

## 3 Conclusion

In summary, inspired by the theoretical simulation predictions, a facile ultrasonic bath strategy was proposed to elaborately integrate the perovskite structured BiFeO_3_ and BiVO_4_ as a unique p–n heterojunction. The well-developed built-in electric field at the heterointerfaces facilitates the local charge redistribution and thus endows the BiFeO_3_ and BiVO_4_ surfaces with local nucleophilic and electrophilic regions, which promote the targeted adsorption and activation of N_2_ and CO_2_ molecules. Besides, the local charge redistribution also contributed to fully exposing the active sites and accelerated electrocatalytic kinetics, which is beneficial for the formation of the C–N bond and generation of the desired *NCON* intermediate. As a result, the BiFeO_3_/BiVO_4_ hybrids exhibit a maximum urea yield rate and FE of 4.94 mmol h^−1^ g^−1^ and 17.18% at −0.4 V *vs.* RHE in 0.1 M KHCO_3_. Besides, the related urea yield rate and FE can be further improved to 5.42 mmol h^−1^ g^−1^ and 20.75% in (Bmim)BF_4_–KHCO_3_ electrolyte. This work proposed an innovative local charge redistribution concept to design urea production catalysts by promoting electrocatalytic C–N bond coupling.

## Author contributions

The manuscript was written through contributions of all authors. All authors have given approval to the final version of the manuscript.

## Conflicts of interest

There are no conflicts to declare.

## Supplementary Material

SC-012-D1SC01467F-s001
