# Supplementary material for: Electrochemical C–N coupling with perovskite hybrids toward efficient urea synthesis
Source: Chem Sci. 2021 Apr 12;12(17):6048–58. doi: 10.1039/d1sc01467f (PMC8098680; doi:10.1039/d1sc01467f)
Supplement: SC-012-D1SC01467F-s001 [file SC-012-D1SC01467F-s001.pdf]

Electronic Supplementary Material (ESI) for Chemical Science.
This journal is © The Royal Society of Chemistry 2021

Supporting information

Menglei Yuan [ab], Junwu Chen [ab], Yiling Bai [cd], Zhanjun liu [c], Jingxian Zhang [ab], Tongkun Zhao [ab], Qiaona Shi [e], Shuwei Li [a], Xi Wang [fg], Guangjin Zhang [abg]*

Experimental Section

Materials

Iron nitrate nonahydrate [$Fe(NO_3)_3 \cdot 9H_2O$, 99.99%], bismuth nitrate pentatydrate [$Bi(NO_3)_3 \cdot 5H_2O$, 99.99%], ammonium metavanadate [NH_4VO_3, 99.9%], ethylene glycol [$C_2H_6O_2$, ≥ 99.5%], phosphoric acid [H_3PO_4, ≥ 85%], sulfuric acid [H_2SO_4, ≥ 85%], iron chloride [$FeCl_3$, 99.9%], diacetylmonoxime [$C_4H_7NO_2$, AR], thiosemicarbazide [CH_5N_3S, 99%] and potassium bicarbonate [$KHCO_3$, ≥ 99.99%] were purchased from Sigma-Aldrich Chemical Reagent Co., Ltd. Water were purified by Millipore system and ethanol was utilized without further purification.

Preparation of rice-like $BiVO_4$ nanoparticles

10 mmol NH_4VO_3 and 10 mmol $Bi(NO_3)_3 \cdot 5H_2O$ were dissolved in 40 mL hot water and 40 mL ethylene glycol, respectively. After that, the NH_4VO_3 solution was slowly dropwised into $Bi(NO_3)_3 \cdot 5H_2O$ solution and vigorously stirred for 1h. Next, the obtained mixed solution were transferred into an ultrasonic bath at 40 °C and further treated for several minutes until generating yellow precipitates. The precipitates were finally washed with abolute ethanol and deionized water to remove any impurities.

Preparation of $BiFeO_3$ nanoparticles

10 mmol $Bi(NO_3)_3 \cdot 5H_2O$ were firstly dissolved in 40 mL ethylene glycol to form a transparent solution. Then, 10 mmol $Fe(NO_3)_3 \cdot 9H_2O$ were directly added into above solution and vigorously stirred several minutes. The formed dark red sol was dried in a vacuum oven at 60 °C and then transferred into a tube furnace and calcined at 550 °C for 2h with a heating rate of 3 °C/min.

Preparation of rice-like $BiFeO_3/BiVO_4$ nanoparticles

The $BiFeO_3/BiVO_4$ hybrids were prepared by a facile ultrasonic bath method. 10 mmol NH_4VO_3 and 10 mmol $Bi(NO_3)_3·5H_2O$ were dissolved in 40 mL hot water and ethylene glycol, respectively. After that, the NH_4VO_3 solution was slowly dropwised into $Bi(NO_3)_3·5H_2O$ solution and vigorously stirred for 1h. Then 40 mL of $BiFeO_3$ precursor were slowly dropwised into the above solution to form suspensions under continuous stirring. The resulting orange precipitates were dried in a vacuum oven at 60 ℃ and then transferred into a tube furnace and calcined at 550 ℃ for 2h with a heating rate of 3 ℃/min.

Characterization

X-ray diffraction (XRD, X'PERT PRO MPD diffractometer, Cu Kα radiation, λ=0.15418 nm, scanned range of 2-90°) was used to identify the crystal structure of all prepared catalysts. Scanning electron microscopy (SEM, JSM-7800F Prime) and transmission electron microscopy (TEM, JEM-2100F) were utilized to investigate the morphology of all samples. The Raman measurements were carried out on a Renishaw Raman Test system (λ=532 nm). Nitrogen adsorption-desorption isotherms were recorded on the nitrogen-adsorption apparatus (AUTOSORB-IQ-XR-C) and specific surface area was calculated based on the Brunauer-Emmett-Teller (BET) equation. X-ray photoelectron spectroscopy (XPS) data were collected by using Krato, AXIS-HS monochromatized Al Kα cathode source of 75-150 W under ultrahigh vacuum. Moreover, the UV-visible adsorption spectra were recorded on a spectrophotometer (UV-2550). H NMR spectra were collected on a superconducting-

magnet NMR spectrometer (Bruker AVANCE III HD 700 MHz).

Electrochemical measurements

All electrochemical characterizations were performed using a CHI 660E workstation coupled with a three-electrode system in a two-compartment cell separated by Nafion 211 membrane. And the Nafion membrane was treated by boiling in ultrapure water for 1 h and heating in H_2O_2 (5%) aqueous solution at 80℃ for another 1 h, respectively. Carbon cloth utilized in this work was purchased from CeTech (W1S1009 type) and treated with the mixture of H_2SO_4:H_2O_2 (1:3 vol.) for 12 h to remove surface impurities. To avoid contamination with nitrogen-containing species in air, electrodes were used either immediately after preparation or kept in vacuum before being used in electrochemical experiments. The prepared catalyst loaded on a piece of pretreated carbon cloth (1×3 cm^2) was used as the working electrode, a graphite rod and Ag/AgCl (saturated KCl electrolyte) were employed as counter electrode and reference electrode, respectively. Potential without iR-compensated were converted to RHE scale via the following equation: E (vs. RHE) = E (vs. Ag/AgCl) + 0.0591 × pH + 0.197 (pH = 6.8 in CO_2-saturated electrolyte and N_2 + CO_2-saturated electrolyte in 0.1 M $KHCO_3$; pH = 8.3 for N_2-saturated electrolyte in 0.1 M $KHCO_3$; pH = 8.0 for Ar-saturated electrolyte in 0.1 M $KHCO_3$). The catalyst ink for working electrode was prepared by dispersing 3.23 mg of catalyst in a mixed solution of 30 μL Nafion (0.5 wt%), 500 μL ethanol and 470 μL water followed by sonication for 30 minutes. Mass loading of 0.3 mg cm^{-2} was used for electrochemical study. All experiments were carried out at room temperature (25℃). To remove the

impurities in the inlet gas, such as NO_X, the prepurification of high-purity N_2 (purity 99.999%) and CO_2 (purity 99.999%) by passing through a saturator filled with 0.05 M NaOH and a saturator filled with 0.05 M H_2SO_4 solution to remove any possible contaminants. Before carrying out all the electrochemical characterizations, the 0.1 M $KHCO_3$ electrolyte solution was purged with CO_2 + N_2 for 30 minutes. Cyclic voltammetry (CV) test was carried out on at a scan rate of 50 mV s^{-1} ranging from 0-0.2 V (vs. RHE). Linear sweep voltammetry (LSV) was also conducted at a scan rate of 5 mV s^{-1}. Chronoamperometric test were then conducted at different potentials and CO_2 + N_2 was continuously fed into the cathodic cell during the experiments. The recycle test was to perform five consecutive cycles of chronoamperometric runs without changing the electrolyte at -0.4 V vs. RHE. Electrochemical impedance spectroscopy (EIS) was conducted at a frequency range from 100 kHz to 1 Hz with a 10 mV AC signal amplitude at -0.4 V vs. RHE on a PAR-STAT 2273 test system. And the electrolyte were saturated with CO_2 and N_2 during EIS tests. The Mott-Schottky plots were measured on an electrochemical workstation (CHI 660E) and the Na_2SO_4 solution were utilized as the electrolyte. A typical three-electrode system was equipped, including the samples (coated on the ITO glass) as working electrode, a Ag/AgCl as reference electrode and Pt foil as counter electrode.

Determination of urea concentration by diacetyl monoxime method

The urea concentration was determined by diacetyl monoxime method [*Clin Chim Acta.,* **1980,** *107,* 3-9]. 5g of diacetylmonoxime (DAMO) and 100 mg of thiosemicarbazide (TSC) were dissolved in distilled water and diluted to 1000 mL,

denoted as DAMO-TSC solution. Then, 100 mL concentrated phosphoric acid was mixed with 300 mL of concentration sulfuric acid and 600 mL distilled water, then 100 mg $FeCl_3$ was dissolved in the above solution, denoted as acid-ferric solution. Typically, 1 mL of the sample solution was removed from the cathodic chamber. Afterwards, 1 mL of DAMO-TSC solution and 2 mL of acid-ferric solution were added into 1 ml of sample solution. Next, the mixed solution was heated to 100 ℃ and maintained at this temperature for 15 min. When the solution cooled to 25 ℃, the UV-Vis absorption spectrum was collected at a wave-length of 525 nm. The concentration-absorbance curve was calibrated using standard urea solution for a series of concentrations. The fitting curve shows good linear relation of absorbance value with urea concentration by three times independent calibration tests.

Calculation of Faradaic efficiency (FE) and urea formation rate

The FE for urea electrosynthesis was defined as the amount of electric charge used for producing urea divided the total charge passed through the electrodes during the electrolysis. Assuming six electrons were needed to produce one urea molecule, the FE was calculated according to the following equation:

$$FE = 6 \times F \times C_{urea} \times V / (60.06 \times Q)$$

The rate of formation of urea was calculated using the following equation:

$$\text{urea yield rate} = C_{urea} \times V / (m_{cat} \times t \times 60.06)$$

Where F is Faraday constant (96485 C mol^{-1}), C_{urea} is the measured mass concentration of urea; V is the volume of the cathodic reaction electrolyte; Q is the quantity of applied charge/electricity; t is the time for which the potential was applied;

m is the mass of catalyst loaded at the carbon cloth.

Determination of NH_3 concentration by indophenol blue method

When tested in 0.1 M $KHCO_3$, the produced NH_3 was spectrophotometrically determined by the indophenol blue method [*Nat Mater.* **2013,** *12*, 836-841]. Typically, 2 mL of the sample solution was removed from the cathodic chamber. Afterwards, 2 mL of 1.0 M NaOH solution containing 5 wt% salicylic acid and 5 wt% sodium citrate was added, followed by 1 mL NaClO solution (0.05 M) and 0.2 mL of an aqueous solution of sodium nitroferricyanide (1 wt%) were added. After standing at room temperature for 2 hours, the UV-Vis absorption spectrum was collected at a wave-length of 655 nm. The concentration-absorbance curve was calibrated using standard NH_4Cl solution for a series of concentrations. The fitting curve shows good linear relation of absorbance value with NH_4Cl concentration by three times independent calibration tests.

Calculation of Faradaic efficiency (FE) and NH_3 formation rate

The FE for NRR was defined as the amount of electric charge used for producing NH_3 divided the total charge passed through the electrodes during the electrolysis. Assuming three electrons were needed to produce one NH_3 molecule, the FE was calculated according to the following equation:

$$FE = 3 \times 0.318 \times F \times C_{NH4Cl} \times V / (17 \times Q)$$

The rate of formation of NH3 was calculated using the following equation:

$$NH_3 \text{ yield rate} = 0.318 \times C_{NH4Cl} \times V / (m_{cat} \times t \times 53.5)$$

Where F is Faraday constant (96485 C mol^{-1}), C_{NH4Cl} is the measured mass

concentration of NH_4Cl; V is the volume of the cathodic reaction electrolyte; Q is the quantity of applied charge/electricity; t is the time for which the potential was applied; m_{cat} is the mass of catalyst loaded at the carbon cloth.

DFT calculations

DFT calculations were performed using Vienna Ab initio Simulation Package (VASP). The exchange-correlation energy was described using the revised Perdew-Burke-Ernzerhof exchange-correlation density functional (PBE) within the generalized-gradient approximation (GGA). A 400 eV plane-wave kinetic energy cutoff was chosen. We created 1 × 1 × 1 supercell to simulate $BiVO_4$ surface and 2 × 2 × 2 supercell to simulate $BiFeO_3$ surface. The atomic positions were fully relaxed until a maximum energy difference and residual force on atoms, respectively, converged to 10^{-5} eV and 0.03 eV $Å^{-1}$ eV and a 20 Å thick vacuum layer was used to avoid the interaction between top and bottom surfaces. The free energy of the electrochemical steps of the reaction were calculated based on the computational hydrogen electrode (CHE) model. The free energies of species were calculated as: $G = E_{DFT} + E_{ZPE} - T\Delta S$, where E_{DFT} was obtained from DFT energy, E_{ZPE} and $T\Delta S$ of adsorbed species were calculated by vibration analysis, whereas the thermodynamic corrections for gas molecules were from standard database.

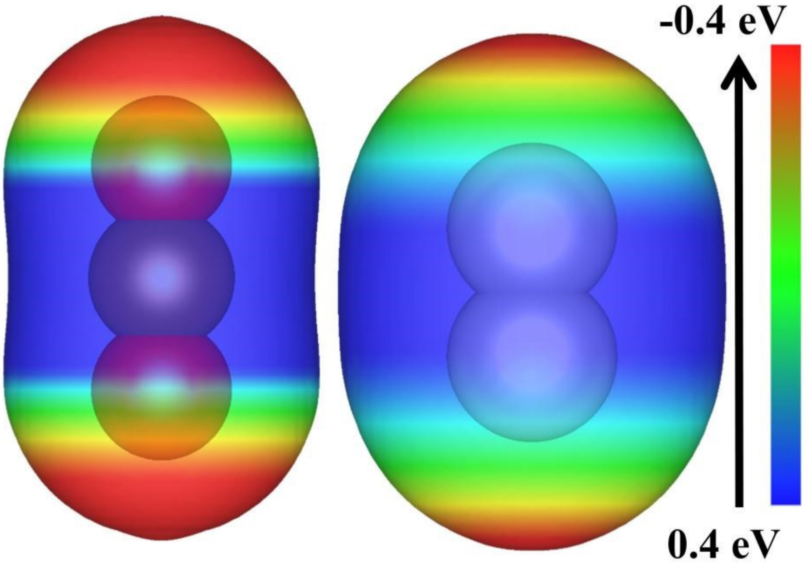

Figure S1. The electron-density isosurface of CO_2 molecule (left) and N_2 molecule (right), the color bar represents the electrostatic potential scale.

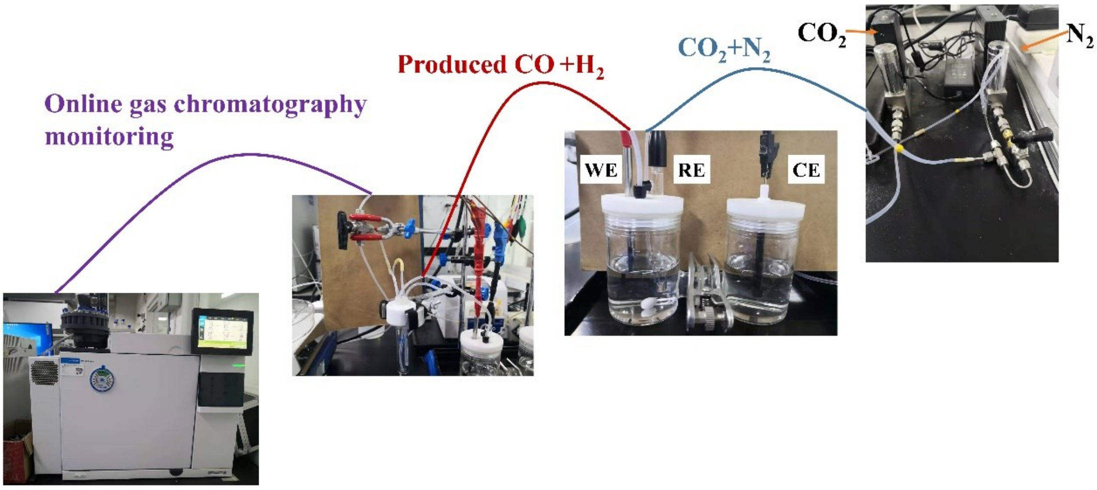

Figure S2. The optical photograph of the three-electrode device for urea electrosynthesis testing.

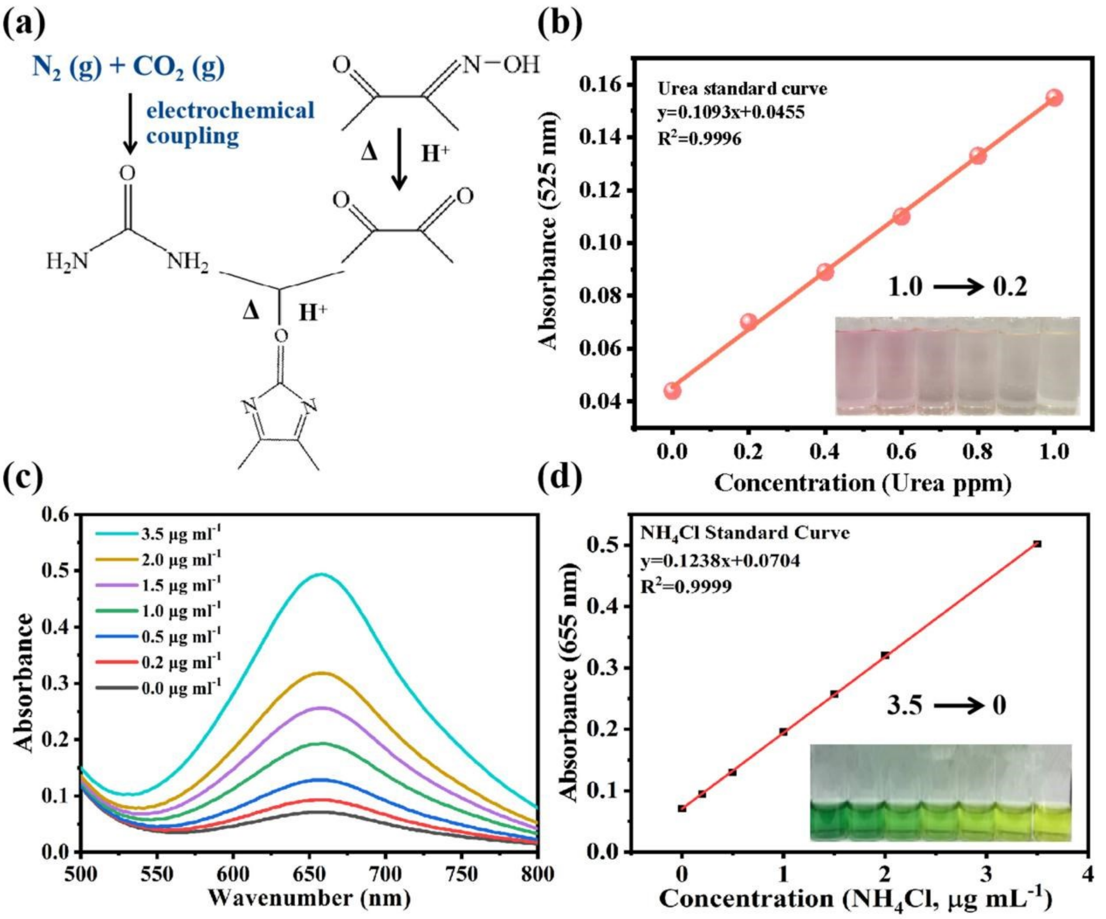

Figure S3. (a) Experimental scheme for the electrochemical synthesis of urea and subsequent determination of the urea concentration generated. Urea detection is based on the diacetyl monoxime method; (b) concentration-absorbance of urea solution with a series of standard concentration (0.2-1.0 μg mL^{-1}) in 0.1 M $KHCO_3$. The absorbance at 525 nm was measured by UV-vis spectrophotometer. The standard curve shown good linear relation of absorbance with urea concentration (y=0.1093x+0.0455, R^2=0.9996); (c) UV-vis curves and (d) concentration-absorbance of NH_4Cl solution with a series of standard concentration (0-3.5 μg mL^{-1}) in 0.1 M $KHCO_3$. The absorbance at 655 nm was measured by UV-vis spectrophotometer. The standard curve shown good linear relation of absorbance with NH_4Cl concentration (y=0.1238x+0.0704, R^2=0.9999).

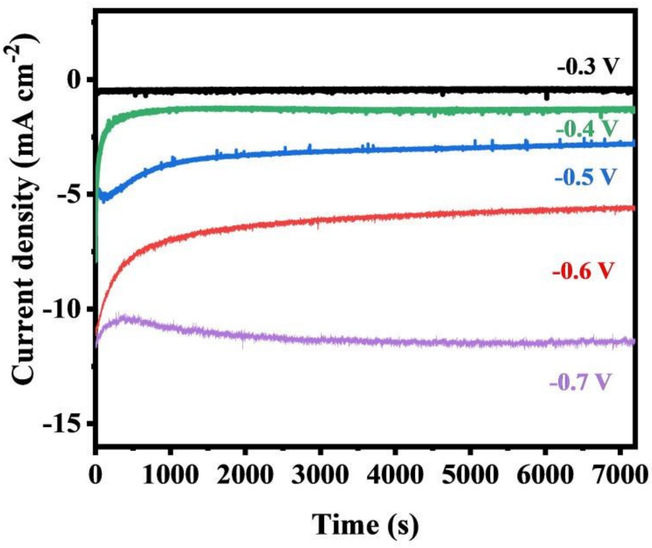

Figure S4. The chronamperometric curves of $BiFeO_3/BiVO_4$ hybrids at various potentials for 2h in N_2 and CO_2-saturated 0.1 M $KHCO_3$ solution.

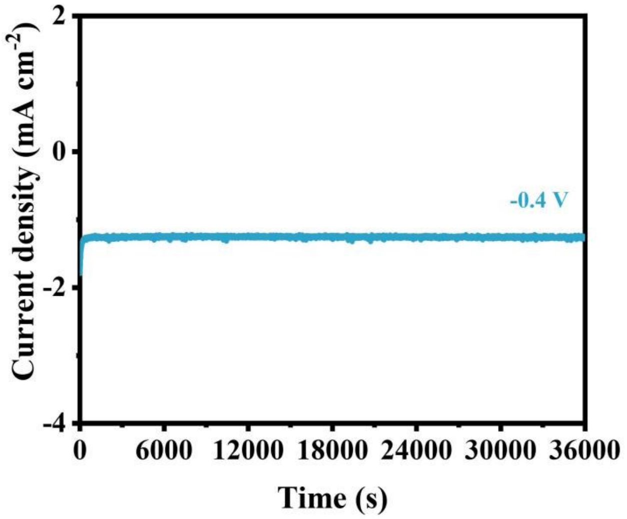

Figure S5. The chronamperometric curves of $BiFeO_3/BiVO_4$ hybrids at -0.4 V vs. RHE for 10h in N_2 and CO_2-saturated 0.1 M $KHCO_3$ solution.

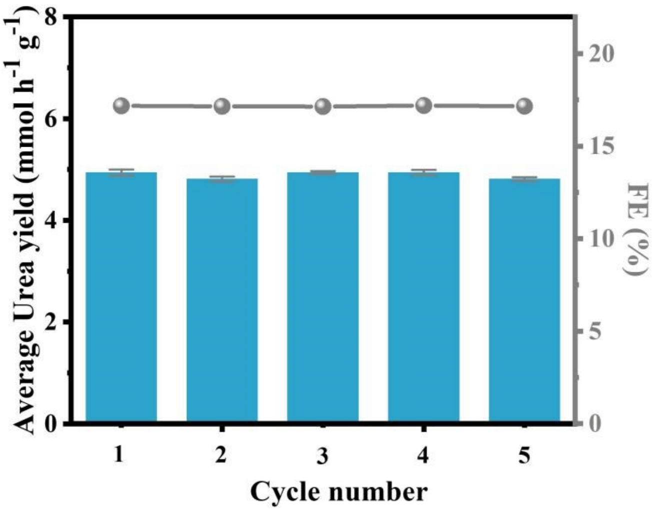

Figure S6. The Faradaic efficiency and urea production rate of $BiFeO_3/BiVO_4$ hybrids at -0.4 V vs. RHE during recycling tests for five times.

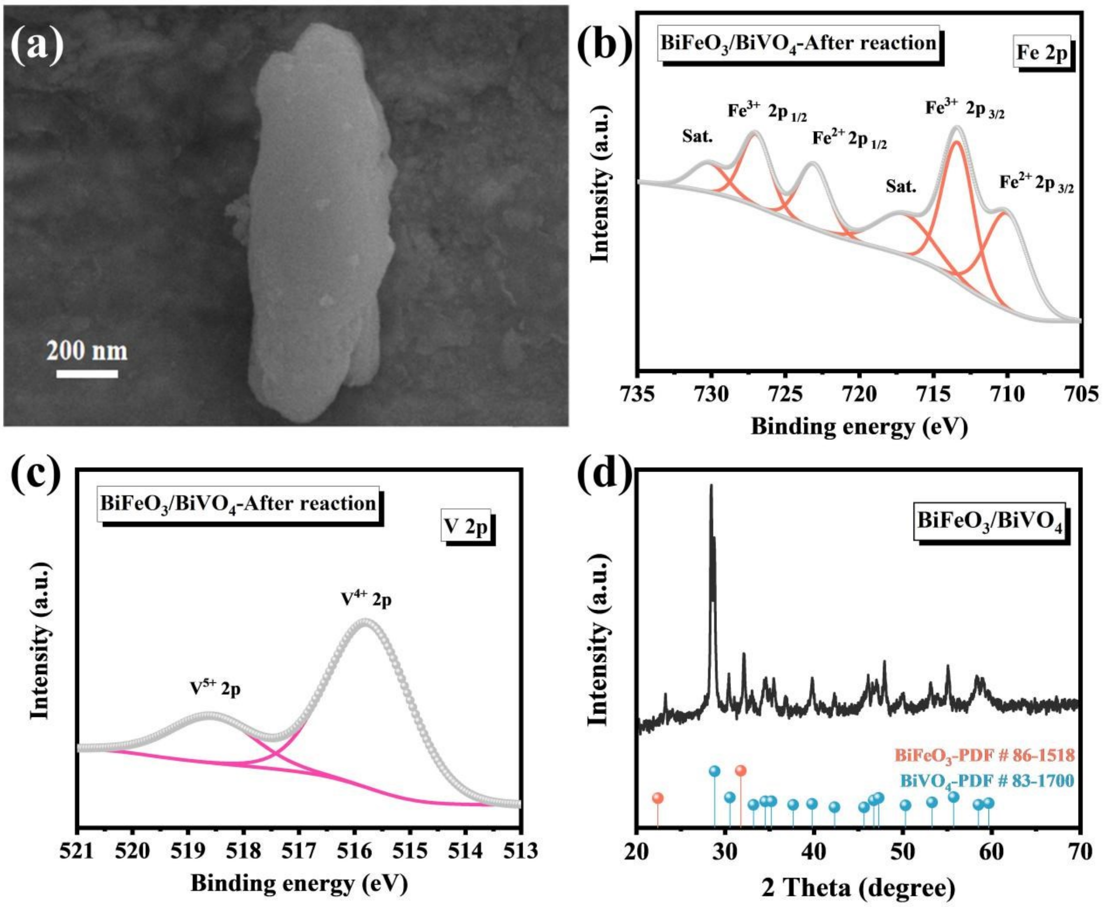

Figure S7. (a) SEM image; (b) high-resolution Fe 2p spectrum; (c) high-resolution V 2p spectrum; (d) XRD patterns of $BiFeO_3/BiVO_4$ hybrids after 10h electrolysis.

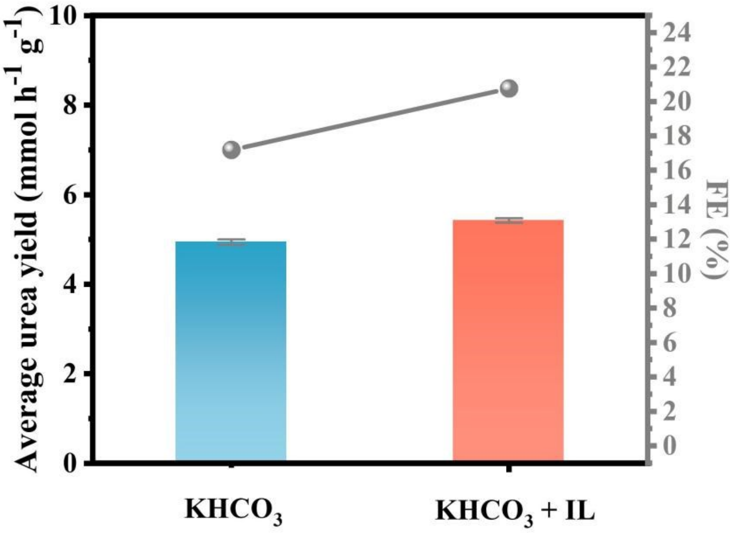

Figure S8. Average urea yield rate and FE in N_2 and CO_2 saturated 0.1 M $KHCO_3$ solution and 0.01 mmol IL + 0.1 M $KHCO_3$ solution (IL = ionic liquid, 1-butyl-3-methylimidazolium tetrafluoroborate).

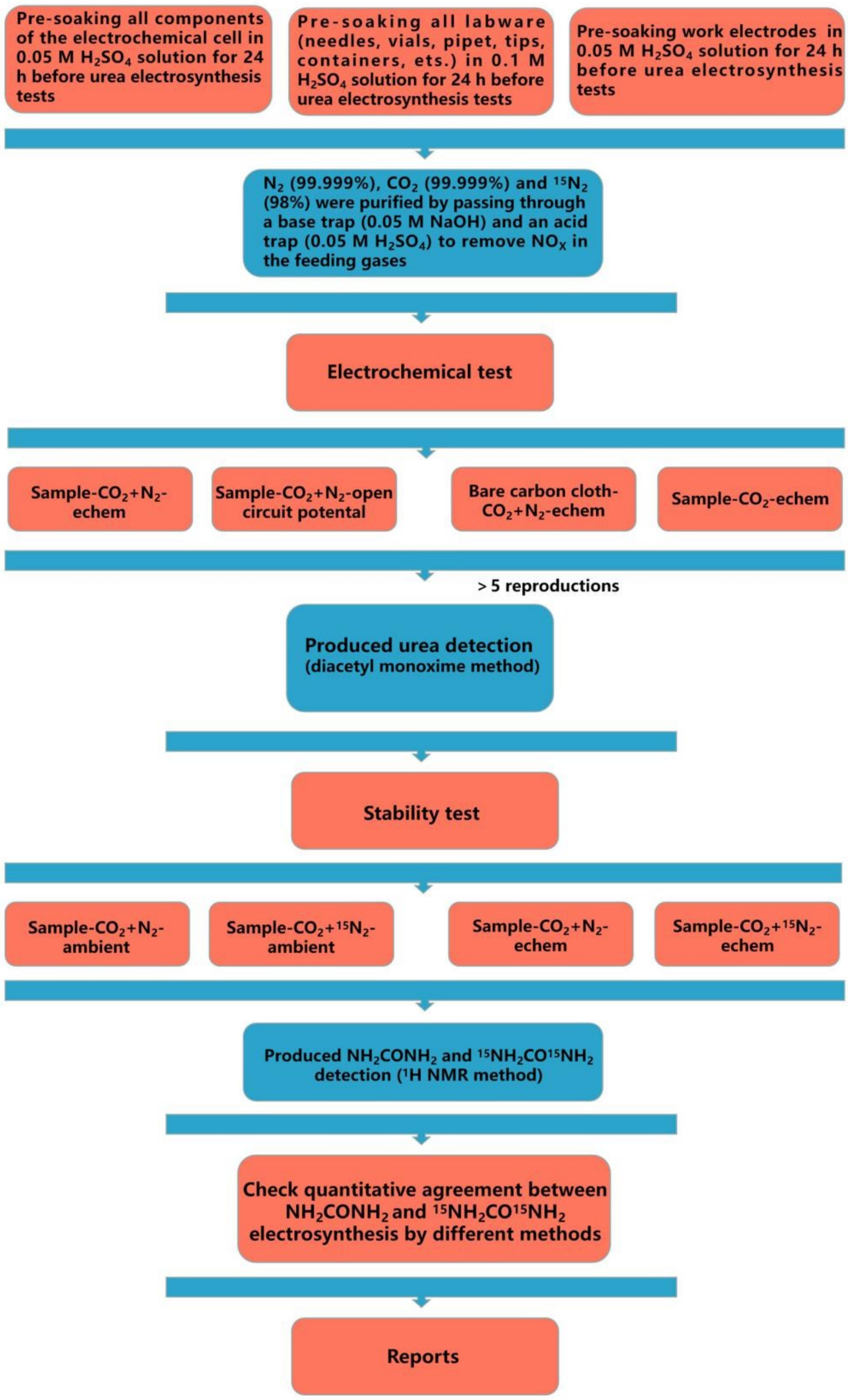

Figure S9. The urea electrosynthesis experimental procedures we utilized in this article.

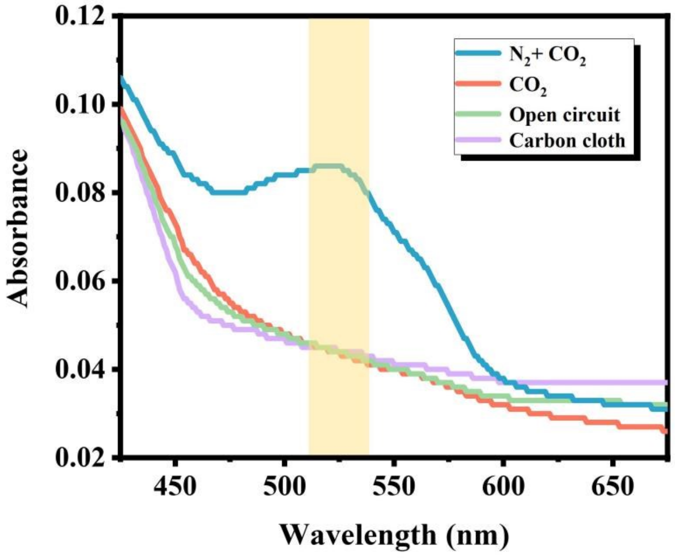

Figure S10. UV-vis spectra of the electrolyte stained with diacetyl monoxime indicator for the bare carbon cloth electrolysis 2h in N_2+CO_2-saturated solution, without and after 2h electrolysis at the potential of -0.4 V in N_2+CO_2-saturated solution, electrolysis 2h in CO_2-saturated solution.

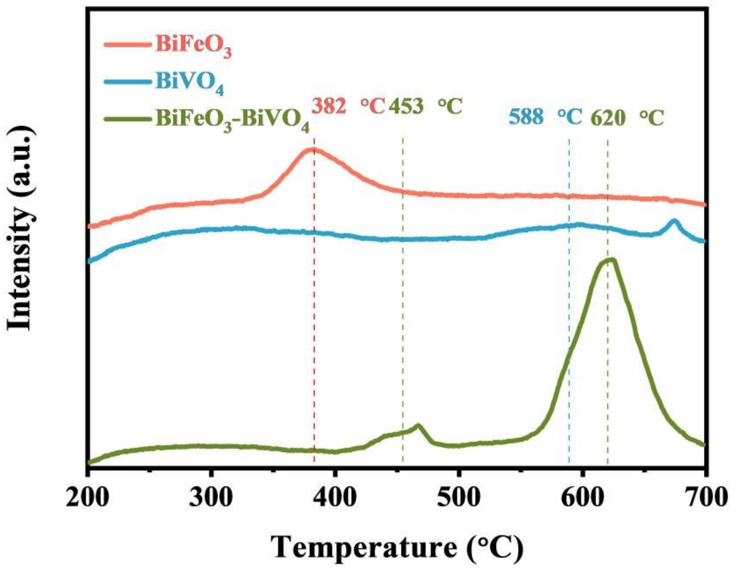

Figure S11. Nitrogen-programmed desorption (N_2-TPD) spectra of $BiFeO_3$, $BiVO_4$ and $BiFeO_3/BiVO_4$ hybrids.

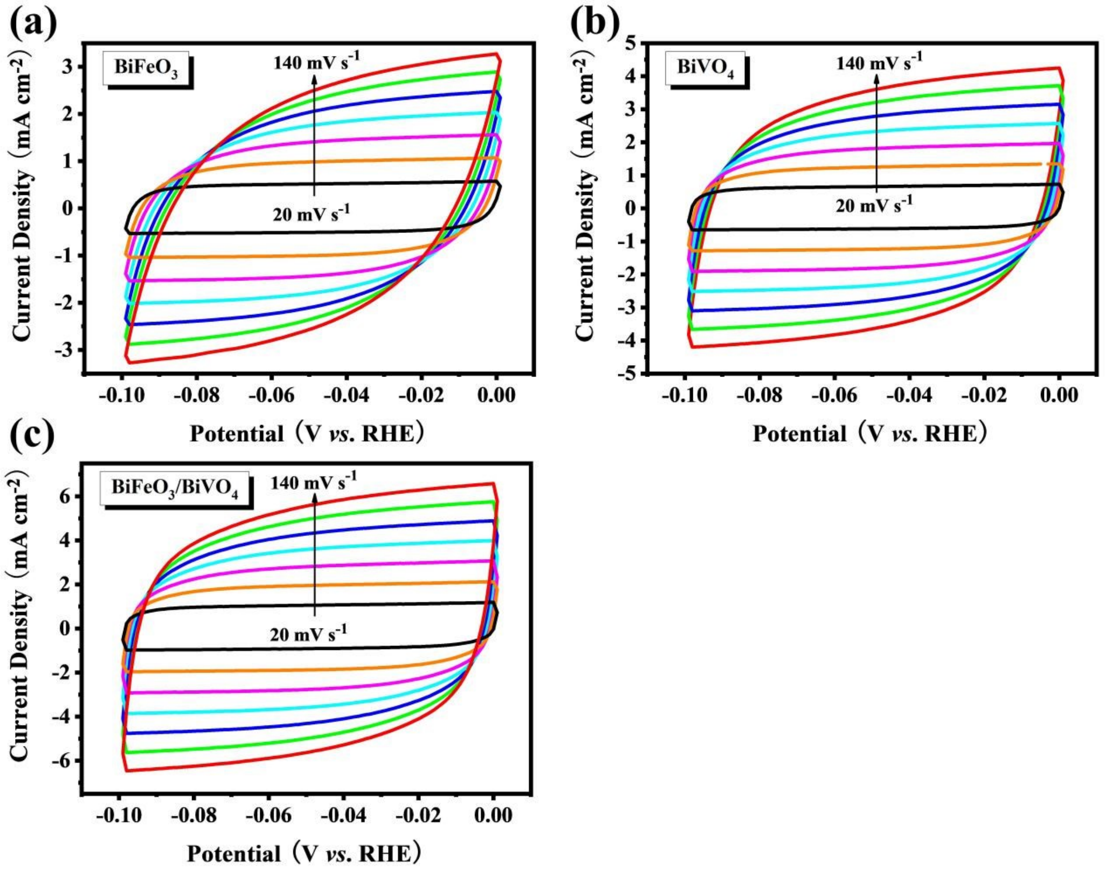

Figure S12. CV curves of (a) $BiFeO_3$; (b) $BiVO_4$; (c) $BiFeO_3/BiVO_4$ with different scan rates from 20 to 140 mV/s.

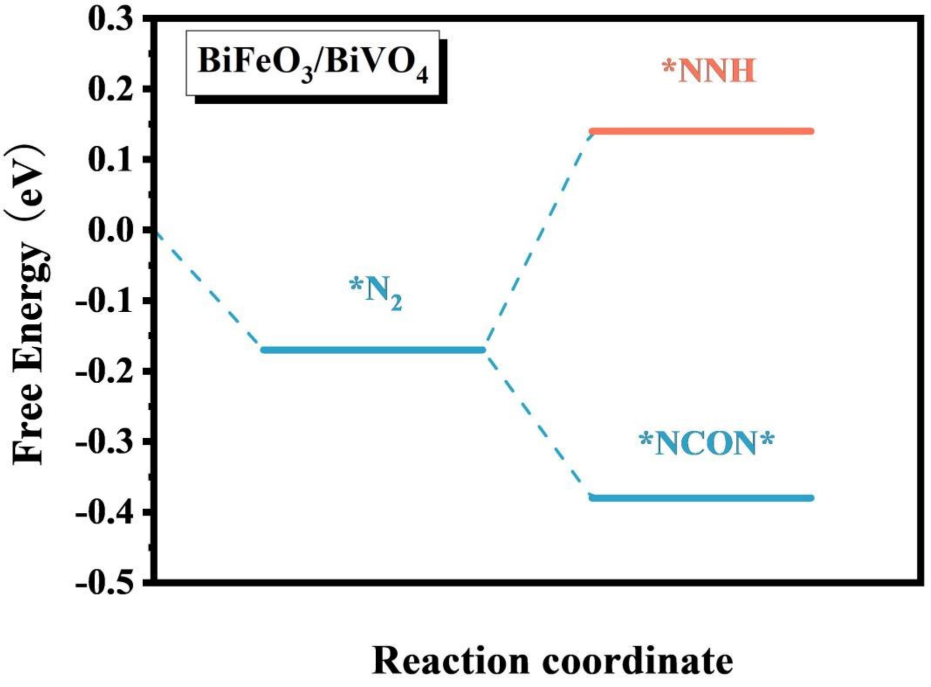

Figure S13. The Free energy diagrams for N_2 adsorption and further activation on the $BiFeO_3/BiVO_4$ surface.

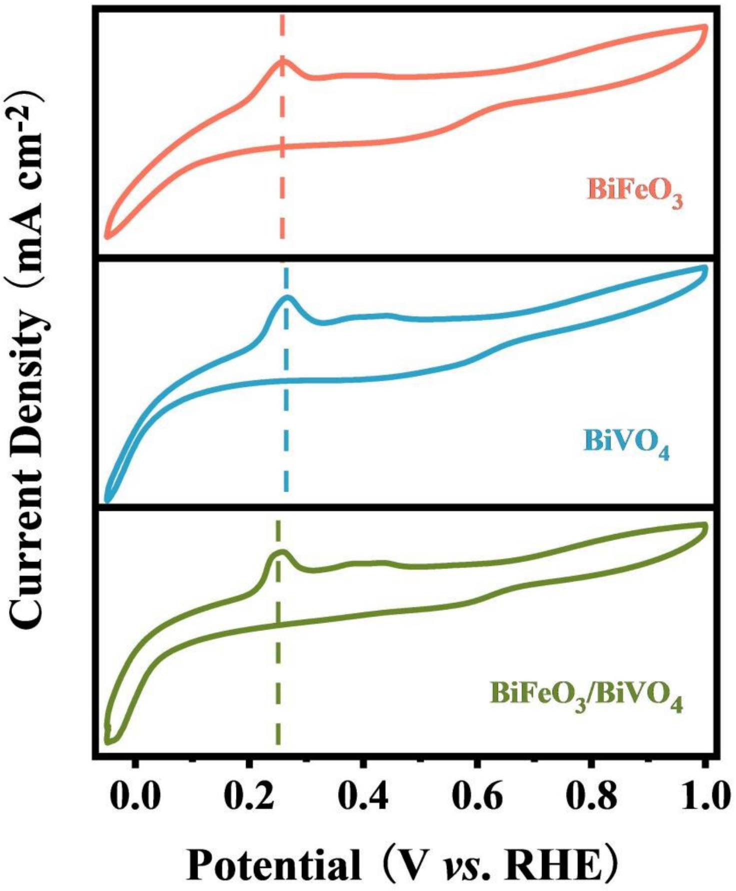

Figure S14. CO-stripping measurements of the $BiFeO_3$, $BiVO_4$ and $BiFeO_3/BiVO_4$ hybrids.

Table S1. Comparison of the electrocatalytic activity of $BiFeO_3/BiVO_4$ hybrids to produce urea through urea electrosynthesis process with previously reported urea electrosynthesis catalysts.

Catalysts	Electrolyte	Potential (V vs. RHE)	Faradaic efficiency	Urea yield rate	Reference
$BiFeO_3/BiVO_4$ hybrids	0.1 M $KHCO_3$	-0.4 V	17.18 %	4.94 mmol h^{-1} g^{-1}	This work
Pd_1Cu_1/TiO_2-400	0.1 M $KHCO_3$	-0.4 V	8.92 %	3.36 mmol h^{-1} g^{-1}	Nat Chem., 2020, 12, 717-724
Te-Pd nanocrystal	0.1 M $KHCO_3$ + 0.01 M KNO_2	-1.1 V	12.2 %	--	Nano Lett., 2020, 20, 8282-8289
